# Supplementary material for: Maritime Paleoindian technology, subsistence, and ecology at an ~11,700 year old Paleocoastal site on California’s Northern Channel Islands, USA
Source: PLoS One. 2020 Sep 17;15(9):e0238866. doi: 10.1371/journal.pone.0238866 (PMC7498104; doi:10.1371/journal.pone.0238866)
Supplement: Attachment — Submitted filename: PLOS One Response to reviewers.docx [file pone.0238866.s001.docx]

Response to Reviewers:

The three reviewers provided important comments for revision or comments that help reinforce some of our original interpretations. Below we highlight our response to each comment of the reviewers. Our responses are noted in red text after the corresponding comment by the reviewer. Reviewers 1 and 2 provided mostly supportive comments and consequently most of our attention focused on the comments from Reviewer 3. 5. Review Comments to the Author

Reviewer #1: Erlandson and his colleagues present data from one of the pre-11,000 BP sites on the northern Channel Islands and its analysis with respect to technology, subsistence, and environment. Their paper expands the available information concerning the early Paleocoastal people who occupied the large channel island that existed at that time. The data and analysis are clearly presented, and I can offer no suggestions for their improvement. I noticed, however, that the LcLaren et al. reference cited on line 67 is missing from the References.

We appreciate this reviewer’s comments. We have added the missing reference to MacLaren’s work on Line 449. We have also updated the publication date to 2020 throughout.

Reviewer #2: This manuscript is an excellent addition to the literature on early human occupation along the Pacific Coast of the Americas. Over the past decade, evidence for a terminal Pleistocene pacific coastal migration and subsequent occupation has grown. Yet much of the evidence comes from relatively small sites that none-the-less suggest an established occupation of groups focused on a variety of opportunistic marine and wetland habitats. The site detailed in this manuscript, SRI-26, was excavated by the authors on Santa Rosa Island and provides additional evidence from which to characterize the early occupations found on a hot spot for terminal Pleistocene and Early Holocene occupation in the New World, the Northern Channel Islands of California.

Although small, the site assemblage provides critical data that informs on seasonal movements and habitat use for early occupants that show adept use of a variety of habitats - some of which are no longer extant due to sea level rise - and species that have since become extinct or are threatened today. While the faunal data from this work do raise questions on accurate representations of Paleocoastal diet within these early sites due to issues of erosion and the Schlepp Effect, the technological assemblage, specifically chipped stone crescents, from SRI-26 again provide a link between island technologies and the Western Pluvial Lakes tradition identified on the mainland. Although the technological assemblages have similarities, the CIA and CIB points from SRI-26 are characteristic of those found in other terminal Pleistocene sites on the island and remain unique to island assemblages. This further discourages the argument that the island assemblages were made by mainland groups, conducting forays to the island for resources.

The data presented support the conclusions. Important for reporting of terminal Pleistocene-aged sites is veracity of the dating methods from secure contexts. The authors have complementary dates from both AMS 14C dates and collagen dates placing one of the site loci within the terminal Pleistocene. Other dates obtained from additional loci are within the early Holocene, but the tables indicate that the faunal data are from the terminal Pleistocene-dated locus. The CIA point identified, though just outside of the intact site deposit is an excellent example of this technology that is considered evidence of terminal Pleistocene occupation. Analysis of the faunal material is reported as are the meat weight calculation used for relative edible meat yield for the shell and bone identified in the site. Again, the authors do note that these totals may be impacted by preservation and representational issues, but what does remains of the food remains suggest birds are an important food source, a trend identified in other terminal Pleistocene sites, but not in island sites dating later in time. Unfortunately, archaeobotanical work at the site did not yield material to discuss exploitation of plants during site occupation, but this is not surprising for these early sites due to preservation issues.

The conclusions presented sum up the findings and suggest that the while the early peoples at this site were considered maritime, the site would have been about 4-6 km back from the coast at the time of occupation. This positioning may have been for targeting migratory waterfowl, and perhaps collecting plant resources, while making trips to the coast and the abundant resources there. This interpretation further supports the notion that preservation of other faunal remains such as shellfish, fish, and sea mammal, which are less abundant than birds, may have been impacted by butchering near to the coast and carrying only meat back to the site. This research provides an excellent addition to the growing body of data showing that island Paleocoastal assemblages are distinct from interior Paleoindian assemblages, and likely date to even earlier in time. The data presented also help to inform on the mobility and subsistence pursuits of the early mobile hunter-gatherer groups present along the Pacific coast and offshore islands during the terminal Pleistocene. This further clarifies which landscape features and regions where additional terminal Pleistocene sites may be identified and sampled, before losing more material to erosional processes.

We thank Reviewer 2 for their important comments and synthesis of the findings. It is good to see that we were on track with our interpretations and the noted limitations of some of our findings.

Reviewer #3: This is a clear and well-supported presentation of results from the archaeological site of CA-SRI-26, an 11.7 ka site on the California Channel Islands with a maritime signature, preserved under a covering of alluvium and exposed to recent investigation by erosion. The site setting, stratigraphy, dating, methods, and evidence of technology and subsistence and their similarities and differences in a wider comparative setting are all set out clearly and to a high standard with all the information one would expect.

The significance of the site is that it is of relatively early date for a site with evidence for use of marine resources and advances knowledge of subsistence and settlement patterns in the Channel Islands for this period in comparison with other known sites, where only stone tools or human bone are present. I think this site is the earliest site anywhere on the Pacific coast (not only on the Channel Islands) with actual archaeological evidence of marine food remains. If that is so, then this point should receive greater emphasis.

We welcome this opportunity to clarify our findings. CA-SRI-26 is one of the earliest sites on the Pacific Coast of the Americas but is not the oldest. Indeed as we note, in the Introduction (see Lines 78-79), occupation of the Channel Islands extends back to 13,000 years ago. Still sites of this early age are exceedingly rare.

There are some minor omissions and errors or inconsistencies, which I set out below.

There are two issues that need to be addressed with some further discussion and detail, and both have to do with the wider significance of this site in a world context and for an international audience who are not necessarily familiar with all the details of the American scene.

The first issue is the nature and duration of settlement at CA-SRI-26. Is it a year-round settlement or a seasonal occupation? The Abstract refers to a 'relatively brief occupation' in winter (Lines 51-52), the text refers to the site as evidence that the people who used it lived on the Islands on a 'year-round basis' (line 341). These seem at least partially contradictory. How good is the evidence for year-round occupation, either at CA-SRI-26 or on the islands generally? Given that there are no land mammals (or none that were hunted) on the islands, and little plant food, at least given what is preserved, is there enough food on the islands to support a decent-size population all year round? It seems that marine resources would have to do the job. Yet the remains at CA-SRI-26 are quite meager. This may be due, as the authors say, to schlepp effects, the distance of the site, 4-6 km, from the sea coast, and the likelihood that processing sites with more abundant marine-resource remains are on the seacoast. If that is so, then such sites would presumably be now well below present sea level. That point might be worth making, along with some assessment of the prospects that such sites might have survived (for example beneath marine sediment) and be discoverable. Are there precedents from later periods or ethnography for island populations capable of sustained self-support based mainly on marine resources? Would food storage be required? Would such populations exist in isolation from populations on the mainland? Otherwise the case for year-round settlement seems to rest on the differences in the lithic assemblages compared to the mainland. There is something here that needs some (brief) elaboration.

We appreciate this point. As for seasonality, the sentence on line 348-350 adds in “other sites” which supports the year-round assertion. We also added a sentence to the abstract to help clarify (see line 54). We have made a slight change in wording to further clarify this point. As for marine foods (and terrestrial plants) offering enough protein, we have added two sentences on lines 350-356 that indicates the importance of marine foods to channel Islanders during the Late Holocene, which fostered high population densities and complex sociopolitical systems. These data demonstrate that there was more than enough protein and carbohydrates from plants to support Paleocoastal peoples on the islands.

This raises the second issue, and perhaps the main one. What is the significance of this site in relation to the wider debates about the coastal colonization hypothesis for the Americas? The authors raise this briefly in the Introduction, and in the summary their main point is that the new evidence refutes the ideas of Balter and Yesner that the island sites are just seasonal visits by mainlanders. Balter is a science journalist and Yesner has long been skeptical that maritime lifeways existed before the mid-Holocene, so I'm not sure that their views are that significant anyway. In any case, seasonal visits to the islands would not necessarily refute the hypothesis of earliest coastal colonization (if that is what Balter and Yesner are claiming). Conversely, year-round maritime occupation of the islands does not seem sufficient to necessarily support the coastal colonization hypothesis (the Islands might have been occupied at a later date than the earliest wave of colonization). The main problem here is the dates. If the NCI are on the kelp highway, as referred to in the text, why are there no sites earlier than about 13 ka, given that we have dates back to about 14.5 to 15 ka elsewhere in the Americas (Cooper's Creek, Monte Verde, etc., which, incidentally. are inland and not marine-based). Are the authors over-interpreting the site to make it seem more significant than is actually the case? Or is there more to be said to put the site findings into the wider context of earliest colonization? I think some further discussion, together with reference to more of the wider literature (Potter et al. comes to mind), is needed

These are important points and we have worked to strengthen our initial argument. First, citing Yesner and Balter is important because these viewpoints have yet to be refuted and we believe our data help put these poorly founded arguments to rest. To clarify the relationship of this site to the peopling of the Americas we have expanded the final paragraph of the paper to include three new sentences on this topic (see line 358). Here we document when the earliest occupations were and clarify the significance of CA-SRI-26 even though it is a few millennia younger than these earliest occupations. We also cite Potter et al. (2018) and their alternative discussion.

Minor Details:

The following references are missing: Peltier 2015, Argus et al. 2014, Davis et al. 2019, McClaren et al. 2019

We have added these missing references and checked the ms. for any additional missing references.
Line 207. the Far West. What region does this refer to?

Changed to western North America. Also changed in two other locations for clarification

Lines 209-210. '...assemblage is smaller than CA-SRI-512 but similar...to the larger collection from CA-SRI-512'. Something wrong here with site numbers

Deleted the second use of “larger” and added “nearby.”

Line 239 'greater faunal diversity', greater than what?

Added “than the shellfish assemblage” and deleted “faunal” to clarify.

Lines 278-279 (and Table 3). birds are 54% and fish 29%, but in line 281 text it is said that birds and fish contributed 95%. Percentage in text looks wrong?

Added “along with marine mammals” to this sentence to clarfify that these categories dominate the assemblage.

6. PLOS authors have the option to publish the peer review history of their article ([what does this mean?](https://nam02.safelinks.protection.outlook.com/?url=https%3A%2F%2Fjournals.plos.org%2Fplosone%2Fs%2Feditorial-and-peer-review-process%23loc-peer-review-history&data=02%7C01%7Crickt%40si.edu%7C2eeac654c417450c675808d83f72d9a0%7C989b5e2a14e44efe93b78cdd5fc5d11c%7C0%7C0%7C637329109812027064&sdata=a%2F9CZ%2FEXwGFdCNUCKUbqd%2FZgDD5EgOI6jjRSom%2BonwY%3D&reserved=0)). If published, this will include your full peer review and any attached files.


If you choose “no”, your identity will remain anonymous but your review may still be made public.


**Do you want your identity to be public for this peer review?** For information about this choice, including consent withdrawal, please see our [Privacy Policy](https://nam02.safelinks.protection.outlook.com/?url=https%3A%2F%2Fwww.plos.org%2Fprivacy-policy&data=02%7C01%7Crickt%40si.edu%7C2eeac654c417450c675808d83f72d9a0%7C989b5e2a14e44efe93b78cdd5fc5d11c%7C0%7C0%7C637329109812027064&sdata=HIDODFBY71%2FQz%2BvkjMlFUgz5BiVXn1tpoZ8Mv0zmVCs%3D&reserved=0).

Reviewer #1: No

Reviewer #2: **Yes:** Amy E Gusick

Reviewer #3: No

[NOTE: If reviewer comments were submitted as an attachment file, they will be attached to this email and accessible via the submission site. Please log into your account, locate the manuscript record, and check for the action link "View Attachments". If this link does not appear, there are no attachment files.]

While revising your submission, please upload your figure files to the Preflight Analysis and Conversion Engine (PACE) digital diagnostic tool, [https://pacev2.apexcovantage.com/](https://nam02.safelinks.protection.outlook.com/?url=https%3A%2F%2Fpacev2.apexcovantage.com%2F&data=02%7C01%7Crickt%40si.edu%7C2eeac654c417450c675808d83f72d9a0%7C989b5e2a14e44efe93b78cdd5fc5d11c%7C0%7C1%7C637329109812037056&sdata=mnQ1NVGzLq8OG80vWPkL21e3AqFKThnoRtSFBkp6wV4%3D&reserved=0). PACE helps ensure that figures meet PLOS requirements. To use PACE, you must first register as a user. Registration is free. Then, login and navigate to the UPLOAD tab, where you will find detailed instructions on how to use the tool. If you encounter any issues or have any questions when using PACE, please email PLOS at [figures@plos.org](mailto:figures@plos.org). Please note that Supporting Information files do not need this step.

*In compliance with data protection regulations, you may request that we remove your personal registration details at any time.* [*(Remove my information/details)*](https://nam02.safelinks.protection.outlook.com/?url=https%3A%2F%2Fwww.editorialmanager.com%2Fpone%2Flogin.asp%3Fa%3Dr&data=02%7C01%7Crickt%40si.edu%7C2eeac654c417450c675808d83f72d9a0%7C989b5e2a14e44efe93b78cdd5fc5d11c%7C0%7C1%7C637329109812037056&sdata=il3co2D93ShjnBbT%2BIgNEKME0xLUyM8GDtFQE9LRMis%3D&reserved=0)*. Please contact the publication office if you have any questions.*
