# Supplementary material for: Maritime Paleoindian technology, subsistence, and ecology at an ~11,700 year old Paleocoastal site on California’s Northern Channel Islands, USA
Source: PLoS One. 2020 Sep 17;15(9):e0238866. doi: 10.1371/journal.pone.0238866 (PMC7498104; doi:10.1371/journal.pone.0238866)
Supplement: Attachment — Submitted filename: PLOS One Response to reviewers 2.docx [file pone.0238866.s002.docx]

Response to Reviewers 2:

Reviewer #3: The authors have satisfactorily addressed all my comments except one, and that is a minor detail, but one that is potentially confusing

I refer to lines 219-220: "The CA-SRI-26 assemblage is smaller than those from CA-SRI-512 and Cardwell Bluffs, but similar
in many respects to the [larger] collection from nearby CA-SRI-512."

My original query about this sentence was not about the use of ‘larger’ but about the fact that the sentence seemed to be comparing two assemblages in such a way as to suggest that they are from two different sites, yet both have the same site label CA-SRI-512. It’s the use of the label CA-SRI-512 that I was querying. Were the authors referring to two distinct assemblages from the same site? Or two different sites? And if two different sites, why do they have the same site record number?

On re-reading the sentence in context with the preceding paragraph, I see where the confusion lies. It's the use of CA-SRI-512 twice in the same sentence. There are three sites: CA-SRI-26, CA_SRI-512, which has a larger assemblage and is nearby, and Cardwell Bluffs (CA-SMI-678 et seq.), which is further away. I think the following wording would avoid the potential confusion: "The CA-SRI-26 assemblage is similar in many respects to the nearby assemblage from CA-SRI-512, though smaller, and similar to the Cardwell Bluffs assemblage."

I now see how I misunderstood the authors' original sentence, and how they in turn have misunderstood my comment and in particular my use of 'numbers', which I used to refer to CA site labels, and they, I think, interpreted as referring to quantities of material. Truly are we divided by a common language!

We welcome the chance to clarify this sentence. We have changed it to the following:

“The CA-SRI-26 assemblage is smaller, but similar in many respects to the assemblages from CA-SRI-512 and, to an extent, Cardwell Bluffs.”
